# Mutations in the efflux regulator gene oqxR provide a simple genetic switch for antimicrobial resistance in Klebsiella pneumoniae

**DOI:** 10.1099/mic.0.001499

**Published:** 2024-09-04

**Authors:** Catherine J. Dawson, Amelia Bartczak, Karl A. Hassan

**Affiliations:** 1 University of Newcastle, Newcastle, Australia; 2 ARC Centre of Excellence in Synthetic Biology, Macquarie University, Sydney, Australia

**Keywords:** *Klebsiella pneumoniae, *efflux upregulation, antibiotic resistance, *oqxAB*, *oqxR*

## Abstract

*Klebsiella pneumoniae* is a pathogen of major concern in the global rise of antimicrobial resistance and has been implicated as a reservoir for the transfer of resistance genes between species. The upregulation of efflux pumps is a particularly concerning mechanism of resistance acquisition as, in many instances, a single point mutation can simultaneously provide resistance to a range of antimicrobials and biocides. The current study investigated mutations in *oqxR*, which encodes a negative regulator of the RND-family efflux pump genes, *oqxAB*, natively found in the chromosome of *K. pneumoniae*. Resistant mutants in four *K. pneumoniae* strains (KP6870155, NTUH-K2044, SGH10, and ATCC43816) were selected from single exposures to 30 µg/mL chloramphenicol and 12 mutants were selected for whole genome sequencing to identify mutations associated with resistance. Resistant mutants generated by single exposures to chloramphenicol, tetracycline, or ciprofloxacin at ≥4 X MIC were replica plated onto all three antibiotics to observe simultaneous cross-resistance to all compounds, indicative of a multidrug resistance phenotype. A variety of novel mutations, including single point mutations, deletions, and insertions, were found to disrupt *oqxR* leading to significant and simultaneous increases in resistance to chloramphenicol, tetracycline, and ciprofloxacin. The *oqxAB*-*oqxR* locus has been mobilized and dispersed on plasmids in many Enterobacteriaceae species and the diversity of these loci was examined to evaluate the evolutionary pressures acting on these genes. Comparison of the promoter regions of *oqxR* in plasmid-borne copies of the *oqxR-oqxAB* operon indicated that some constructs may produce truncated versions of the *oqxR* transcript, which may impact on *oqxAB* regulation and expression. In some instances, co-carriage of chromosomal and plasmid encoded *oqxAB-oqxR* was found in *K. pneumoniae*, implying that there is selective pressure to maintain and expand the efflux pump. Given that OqxR is a repressor of *oqxAB*, any mutation affecting its expression or function can lead to multidrug resistance. This is in contrast to antibiotic target site mutations that must occur in limited sequence space to be effective and not impact the fitness of the cell. Therefore, *oqxR* may act as a simple genetic switch to facilitate resistance via OqxAB mediated efflux.

## Introduction

In 2019, antimicrobial resistance (AMR) was associated with approximately 4.95 million deaths worldwide, 1.27 million of which were directly attributed to bacterial AMR, surpassing mortality from diseases such as human immunodeficiency virus infection and malaria [1]. *Klebsiella pneumoniae* is a pathogen of great concern in the global rise of AMR, with 44% of countries in the WHO European region reporting resistance rates of 50% or above [2]. *K. pneumoniae* has a remarkable capacity to acquire resistance, and has been implicated as a reservoir for spreading AMR genes with other clinically relevant pathogens [3].

Bacteria employ a number of resistance mechanisms to overcome antibiotic treatment, one of which is export of antimicrobials from the cell via efflux pumps. *K. pneumoniae* possess a number of intrinsic efflux pumps, major contributors being the RND-family AcrAB and OqxAB pumps that associate with TolC [4]. The OqxAB-TolC pump confers resistance to a range of antibiotics and biocides, including quinolones, tigecycline, ciprofloxacin, nitrofurantoin, tetracycline, benzalkonium chloride, triclosan and SDS [5,6]. Genes encoding OqxAB are natively located in the chromosome of *K. pneumoniae*, but were first discovered in a conjugative plasmid in *Escherichia coli* [7]. Sequencing and phylogenetic analysis has determined that the *oqxAB* operon probably originated from *K. pneumoniae* and has been mobilized by IS26-like transposable elements [8,9]. Transcription of *oqxAB* is influenced by the global regulator RamA, and by two regulators encoded either side of the *oqxAB* operon [10]. These are *rarA*, encoded upstream of *oqxAB* which activates expression, and *oqxR*, encoded downstream and involved in repressing *oqxAB* expression [4,11]. Mutations in *oqxR* have been reported to increase expression of the *oqxAB* pump, leading to increased AMR [4,6, 11,13].

The following study reports novel mutations found in *oqxR* in *K. pneumoniae* that result in increased resistance to antimicrobial substrates of OqxAB and examines the broader distribution of the *oqxAB-oqxR* operon in genomes of the *Enterobacteriaceae*. A mutation at almost any site in the *oqxR* gene that introduces a frameshift or changes a functionally important amino acid residue, and therefore disrupts its activity as a repressor, allows for increased *oqxAB* expression and increased resistance. Consequently, *oqxR* may act as a simple genetic switch to facilitate multidrug resistance, which may be particularly important in the convergence of hypervirulent and multidrug-resistant *K. pneumoniae* strains.

## Methods

### Bacterial culturing and strains

Culturing was performed in cation adjusted Mueller-Hinton (MH; Oxoid) or Lennox-LB (LLB; 10 g tryptone, 5 g yeast extract, 5 g NaCl) medium where indicated. Hyper-mucoid strains were grown at 25 °C to reduce capsule production, while all other strains were grown at 37 °C unless otherwise stated. The strains used are listed in Supplementary Material 1 .

### Mutant generation

Resistant mutants were generated in *K. pneumoniae* strains by plating high-density cultures on selective media. Strains of *K. pneumoniae* were streaked on LLB and incubated overnight. Cells were resuspended from plates in 1× PBS and adjusted to ~1.7×10^10^ c.f.u. ml^–1^ (OD_600_=20). A 25 µl spot corresponding to ~4×10^8^ c.f.u. was transferred to an LLB plate containing 100 µM 2,6-diaminopimelic acid, allowed to dry in a biosafety hood, then incubated at 37 °C for 1 h. Following incubation, spots were scraped from the plate and streaked onto LLB plates containing 30 µg ml^−1^ chloramphenicol, then incubated overnight. Single colonies were cultured directly from plates under chloramphenicol selection and genomic DNA was extracted for whole genome sequencing.

### DNA extraction and whole genome sequencing

Genomic DNA was extracted from liquid cultures using the DNeasy UltraClean Microbial kit (Qiagen). Briefly, 1 ml of overnight culture was used as input and an additional incubation was performed in solution SL (Qiagen) at 70 °C for 10 min prior to vortexing. The rest was performed as per the manufacturer’s instructions. Purity was assessed by nanodrop and concentration quantified by Qubit using the 1× dsDNA Broad Range assay (Thermofisher). Samples were sent for Illumina paired short-read sequencing at SeqCentre (Pittsburgh, USA) using the 200 Mb service. SeqCentre’s methods were as follows: sample libraries were prepared using an Illumina DNA Prep kit and IDT 10 bp UDI indices, and sequenced on an Illumina NextSeq 2000, producing 2×151 bp reads. Demultiplexing, quality control and adapter trimming were performed with bcl-convert v3.9.3.

### Data processing and mutation prediction

Quality assessment of raw reads was performed using fastQC v0.12.1 [14] with default settings. Reads were filtered using fastp v0.23.2 [15] with the following parameters: reads were trimmed for adapter sequences (R1: TCGTCGGCAGCGTCAGATGTGTATAAGAGACAG, R2: GTCTCGTGGGCTCGGAGATGTGTATAAGAGACAG), 15 bases were trimmed from the front of reads, four from ends, and poly-G tail trimming and poly-X trimming from the 3′ end of reads were also enabled. Mutation prediction was performed using Breseq v0.36.1 [16] using default parameters. Reference sequences were KP6870155 [17], ATCC43816 (Chr: NZ_CP064352), SGH10 (Chr: NZ_CP025080, Plasmid: NZ_CP025081) and NTUH-K2044 (Chr: NC_012731, Plasmid: NC_006625).

### Minimal inhibitory concentration assays

MIC assays were conducted according to EUCAST standards [18]. Briefly, twofold serial dilutions of antibiotics (chloramphenicol, tetracycline and ciprofloxacin) were set up in a 96-well plate (Corning) in cation-adjusted MH. Cultures were prepared by resuspending 3–5 colonies from fresh streak plates in cation-adjusted MH and diluted to reach a final inoculum of 5×10^5^ c.f.u. ml^−1^ in each well. Plates were incubated at 37 °C with shaking (500 r.p.m.) and OD_600_ read every 10 min in a LogPhase 600 plate reader (Agilent). Three biological replicates, each with three technical replicates, were performed.

### Mutation rate determination and generation of mutant pools

Resistance mutation rate from a single exposure to above MIC antimicrobials was determined by plating a high-density culture of *K. pneumoniae* on selective agar plates. KP6870155 was streaked over the entire surface of an MH plate and incubated overnight. Cells were resuspended from plates in 1× PBS and adjusted to ~1.7×10^10^ c.f.u. ml^–1^ (OD_600_=20). A 30 µl aliquot (~5×10^8^ c.f.u.) was spread onto 15 cm diameter MH agar plates containing antibiotic at 4× MIC of the WT strain (12 mg l^−1^ chloramphenicol, 4 mg l^−1^ tetracycline, 0.12 mg l^−1^ ciprofloxacin). Concurrently, the input culture was diluted 1×10^−7^ and 100 µl was plated on non-selective agar to determine c.f.u. ml^–1^. Plates were incubated overnight, protected from light, for 24 h. Plates were then imaged and c.f.u. counted. To determine if resistance was specific to the antibiotic on which mutants were generated, colonies were replica plated in triplicate onto the same panel of antibiotics (chloramphenicol, tetracycline, ciprofloxacin) using sterile filter paper and incubated for a further 24 h protected from light. Plates were imaged, and images were aligned, pseudo-coloured and overlayed using Fiji [19].

### Analysis of the *oqx* operon on plasmid sequences

The *oqx* operon sequence from KP6870155 (from *rarA* to *oqxR*) was queried against the NCBI nucleotide database [20] using blastn and filtered for results that had percentage identity and query coverage of >90% (date accessed 11 March 2023). Given the large number of *Klebsiella* chromosomal sequences in the database, searches were also performed with exclusion of hits to *Klebsiella* (taxid: 570) sequences to allow a greater diversity of variants from other taxa to be identified. This resulted in 321 sequence hits which were exported to a hit-table. These were filtered for hits with an alignment length >4900 nt. Using a custom script, ‘entrez_ncbi_gene_slice.sh’, hit sequences including 10 000 bp upstream and downstream (or when this was not possible, the longest available sequence) were downloaded from the NCBI database utilizing the entrez-direct v16.2 tools [21]. Downloaded sequences were converted to fasta format using the any2fasta.pl script (https://github.com/tseemann/any2fasta) and concatenated into a single multifasta file. CD-hit-est v4.8.1 was run on the concatenated sequences with default settings categorizing sequences into 188 clusters [22]. A representative sequence from each cluster was extracted and re-annotated with Prokka v1.14.6 to ensure consistency in annotations [23]. Splitting the sequences into four subsets, Clinker v0.0.27 was used with default settings to create sequence similarity graphs [24]. Graphs were manually inspected and representative sequences selected based on any divergence from the canonical insertion sequence (IS)-flanked *oqxAB-oqxR* operon previously reported for pOLA52 [7]. Sequences from *K. pneumoniae* were manually selected. From these representative sequences a focused similarity graph was constructed using Clinker.

### Alignment and prediction of *oqxA* and *oqxR* promoter sequences

A region inclusive of the gene and 200 bp upstream of the annotated coding sequence was selected. Sequences were aligned using MAFFT v7.515 [25] with the default L-INS-i parameters. Alignments were visualized using Unipro UGENE [26]. Promoter sequences were predicted using BPROM on the Softberry online webserver (http://www.softberry.com/ accessed 14 August 2023).

### Identification of Tn*6010* in sequences

The sequence for Tn*6010* was obtained from pOLA52 (GenBank ID: EU370913.1) as reported by Norman *et al*. [8]. A custom database was built from the selected sequences using Blast v2.12.0, and the Tn*6010* sequence was then queried against the database using default settings.

### Data availability

Files and custom scripts used in data analysis are available from the GitHub repository: https://github.com/Cat-Jane/Kp_oqxR_mutants. Genome sequence read data were deposited in the GenBank sequence read archive under accession numbers: SRS22372387–SRS22372402.

## Results

### Mutations in *oqxR* promote resistance to chloramphenicol

When constructing *K. pneumoniae* mutants for other projects, we noted a high frequency of false positive antibiotic-resistant colonies. To identify the cause(s) of spontaneous resistance, mutants were generated by plating *K. pneumoniae* KP6870155 at high culture densities on chloramphenicol selective media. Six colonies were selected and subjected to whole genome sequencing. Short reads of the chloramphenicol-resistant mutants were assessed for mutations relative to their ancestral parent strain using Breseq [16]. In all resistant strains, mutations were present in the *oqxR* gene. A variety of mutations were observed including SNPs, 24 bp duplications and a 179 bp deletion (Table 1). To determine if mutations in *oqxR* were specifically selected in strain KP6870155 or whether similar mutations would be selected in other strains, we isolated chloramphenicol-resistant mutants of three hypervirulent strains of *K. pneumoniae*, ATCC 43816, SGH10 and NTUH-K2044, using the same approach. The genomes of two resistant colonies from each strain were sequenced, and it was found that they all carried a mutation in *oqxR* (Table 1). As OqxR is a known negative regulator of the genes encoding the OqxAB efflux pump, it was hypothesized that these mutations were leading to upregulation of the pump to confer resistance. To investigate this further, three KP6870155 strains with distinct *oqxR* mutations were chosen for further analyses: a single amino acid change (A32D), a 24 bp duplication (24dup) and a point mutation leading to a premature stop codon (C100*).

**Table 1.** Mutations in *oqxR* in chloramphenicol-resistant *K. pneumoniae*

Strain	Position	*oqxR* mutation	OqxR change
KP6870155	1 169 748	C→A	A32D (GCC→GAC)
KP6870155	1 169 748	C→A	A32D (GCC→GAC)
KP6870155	1 169 859	(CAACGGCTCAATTCATCTTGGCCG)_1→2_	24 bp duplication → insertion of LGRNGSIH
KP6870155	1 169 953	C→A	C100* (TGC→TGA)
KP6870155	1 169 859	(CAACGGCTCAATTCATCTTGGCCG)_1→2_	24 bp duplication → insertion of LGRNGSIH
KP6870155	1 169 843	T→C	S64P (TCA→CCA)
ATCC 43816	5 327 417	G→C	A32P (GCC→CCC)
ATCC 43816	5 327 529	(CAACGGCTCAATTCATCTTGGCCG)_1→2_	24 bp duplication → insertion of LGRNGSIH
SGH10	1 174 013	Δ179 bp	51RDGIIV →51QIGCR*
SGH10	1 173 875	G→T	R5L (CGC→CTC)
NTUH-K2044	4 041 038	(ATGAATTGAGCCGTTGCGGCCAAG)_1→2_	24 bp duplication → insertion of LGRNGSIH
NTUH-K2044	4 041 038	(ATGAATTGAGCCGTTGCGGCCAAG)_1→2_	24 bp duplication → insertion of LGRNGSIH

* indicates stop codon. Underlines highlight DNA bases that were mutated within the codon leading to an amino acid change.

### *oqxR* mutants have increased tolerance to a range of antimicrobials

**Fig. 1.**
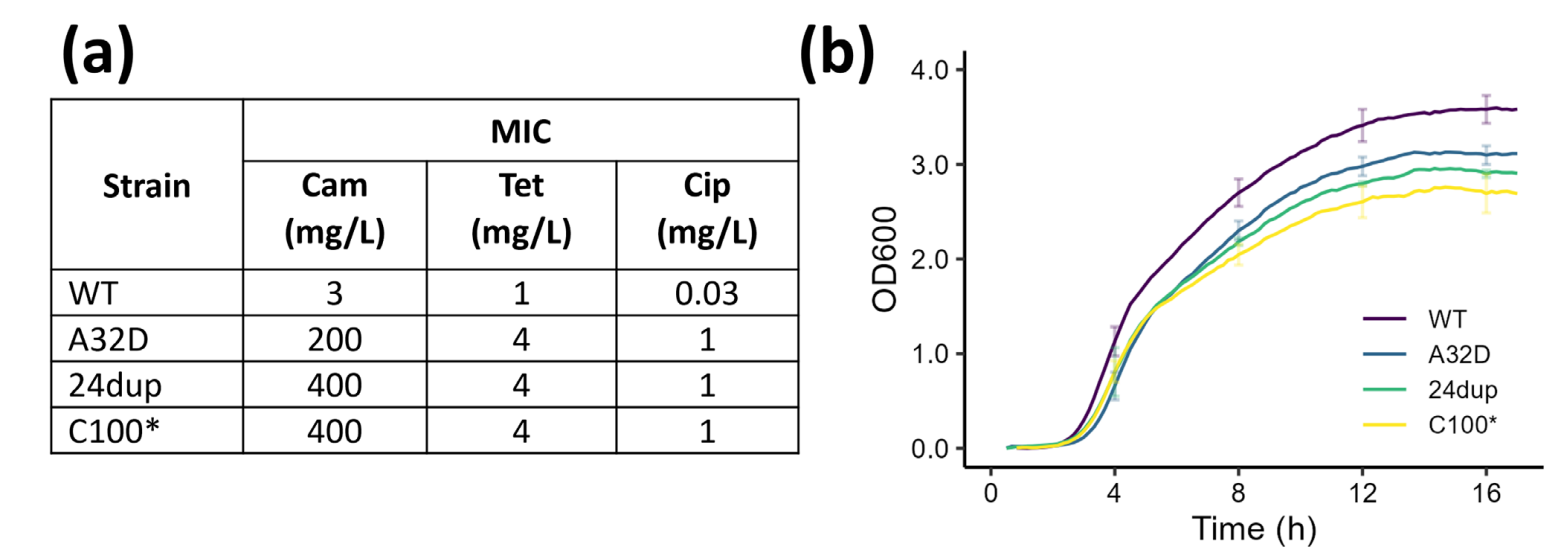
(a) MIC of KP6870155 WT and *oqxR* mutant strains. (b) Growth curves of KP6870155 WT and various *oqxR* mutants, conducted in Muller-Hinton media. Error bars represent sd, average of three biological replicates.

The MICs of several antibiotics for the selected *oqxR* mutants were tested to determine if the mutations impacted resistance. Chloramphenicol, tetracycline and ciprofloxacin are known substrates of the OqxAB efflux pump and each fall into a different antibiotic class. The mutants were selected on chloramphenicol and, as expected, each showed high resistance to this antibiotic with the A32D mutant at 66×, and the 24dup and C100* mutants at 133× the MIC of the parental strain (Fig. 1a) . The difference in MIC was less pronounced for tetracycline with all mutant strains showing resistance at 4× the MIC of the parental strain. Ciprofloxacin MICs were 33× higher than the parental strain for all mutants. These values are classed as resistant in reference to EUCAST susceptibility breakpoints, noting that tetracycline has been removed as a recommended agent for treatment in *Enterobacterales* in the 2023 revision [27]. There were no obvious differences between the strains in the absence of selection in terms of exponential growth rates in MH medium, but the *oqxR* mutants did appear to have a lower stationary phase density (Fig. 1b) .

### Mutation rate determination

**Fig. 2.**
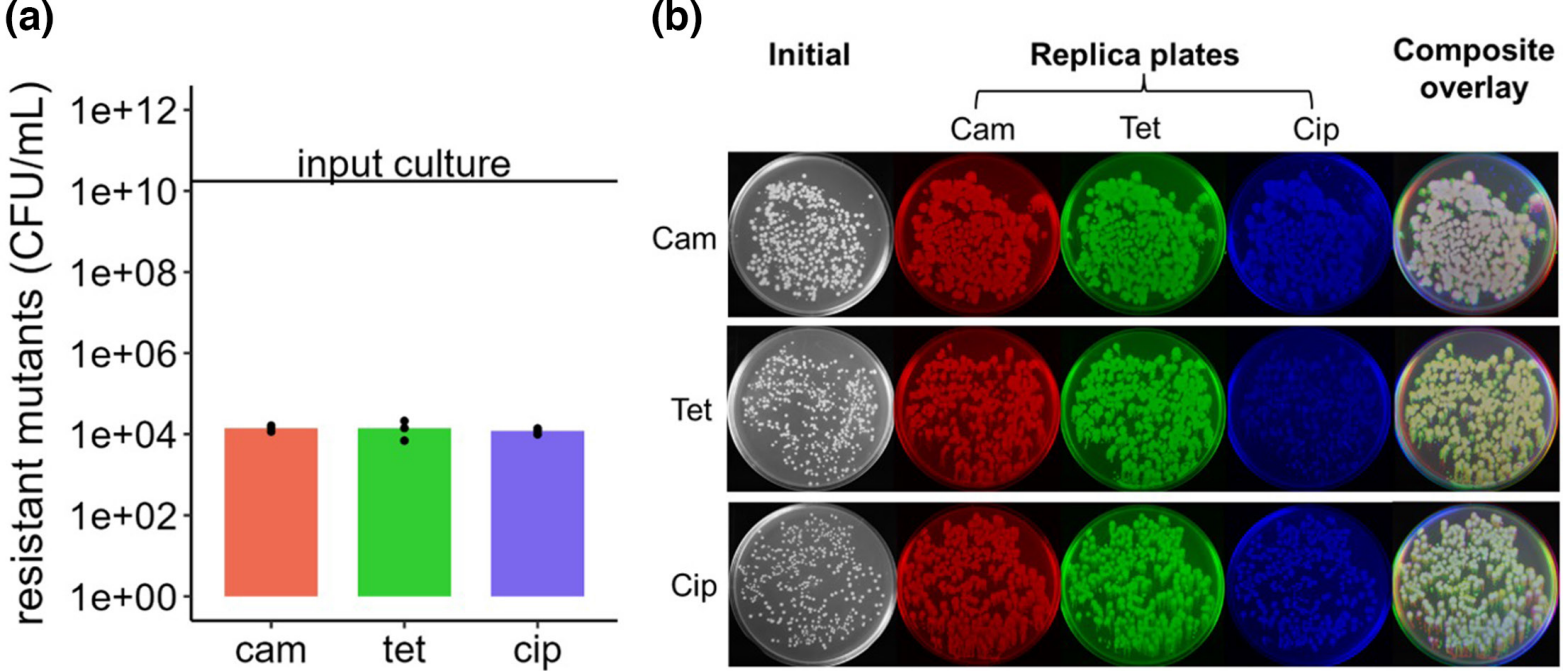
**(a**) Number of resistant mutants that arose from a single exposure to antibiotic selection at 4× the MIC, relative to the WT input culture of 1.74×10^10^ c.f.u. ml^−1^. (**b**) Replica plates of resistant mutants across different antibiotics taken from the initial plate. Images have been pseudo-coloured and overlayed in the final panel.

To determine the rate of antibiotic-resistant mutants selected by one exposure to antibiotic, high-density cultures were plated on media containing 4× MIC of chloramphenicol, tetracycline and ciprofloxacin, and the number of resistant c.f.u. relative to the input were counted. It was also of interest whether different antibiotics would select for greater numbers of mutants than others. From an input culture of 1.74×10^10^ c.f.u. ml^−1^ (sd=1.94×10^9^), a similar number of mutants arose under selection for each antibiotic at ~1×10^4^ c.f.u. ml^–1^ (Fig. 2a), suggesting there was no significant difference between the antibiotics tested in terms of promoting mutant selection at the concentrations used. To help distinguish between mutations in *oqxR-oqxAB* and other mutations, such as tetracycline and ciprofloxacin target site mutations [28,29], colonies that arose on the first selective plate were replica plated onto other antibiotics. Resistance to all antibiotics is indicative of a multidrug-resistant phenotype like that conferred by OqxAB, whereas antibiotic-specific resistance could have arisen through target site mutation. Almost all colonies (>99%) were also able to grow on the other antibiotics tested, suggesting that resistance may be due to efflux pump upregulation rather than a specific target modification or enzymatic antibiotic inactivation (Fig. 2b).

### Sequence conservation of the *oqxAB-oqxR* operon between chromosome and plasmids

**Fig. 3.**
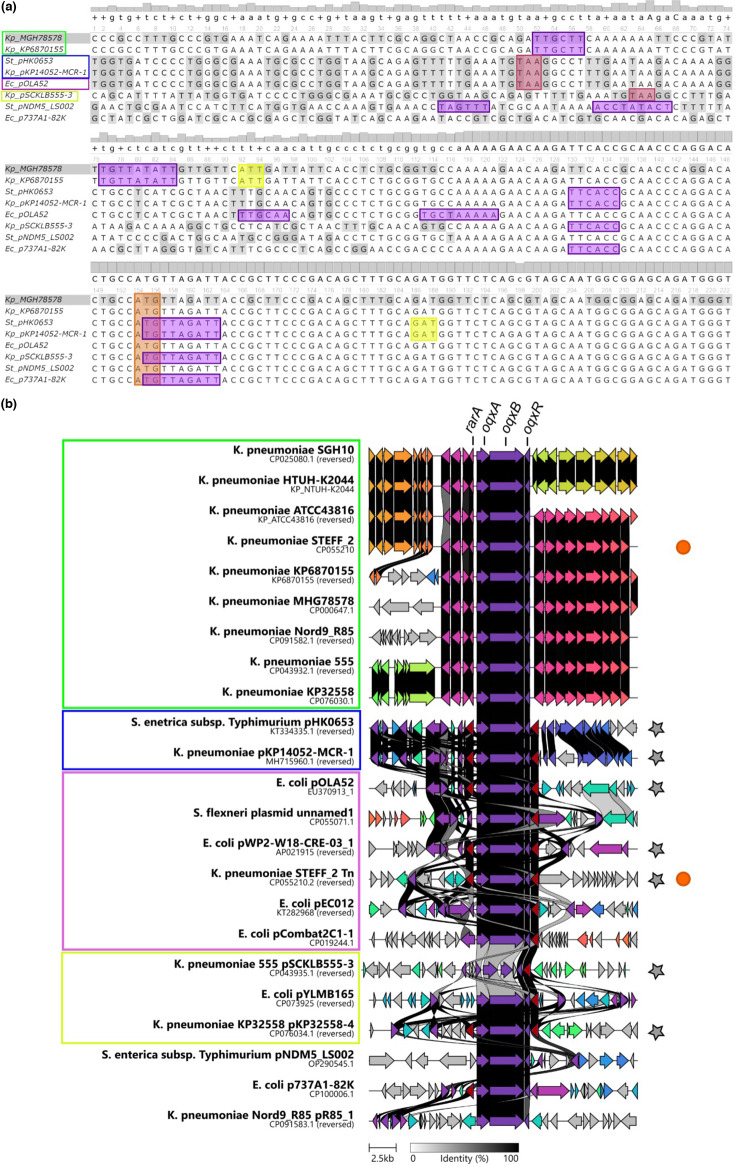
Comparison of *oqxAB-oqxR* sequences from various *Enterobacteriaceae*. (a) Alignment of the *oqxR* promoter region in chromosomal and plasmid sequences. A representative sequence was chosen from each promoter grouping; the coloured boxes correspond to plasmids in (b) that had the same promoter region. The *K. pneumoniae* MGH78578 and St pHK06-53 sequences published by Chan *et al*. [30] are included for comparison. Highlighted are the predicted −35 and −10 promoter sequences (purple), transcription start site (yellow), transposon stop codon (red) and *oqxR* start codon (orange). Kp=*K. pneumoniae*, St=*Salmonella typhimurium*, Ec=*E. coli*. (**b)** Comparison of *oqxAB-oqxR* sequences and their genomic context. The IS sequences are shown in dark red. Coloured boxes indicate sequences with identical *oqxR* promoter regions and how they relate to (a). Sequences that contain Tn*6010* are indicated by a grey star, and sequences from the same chromosome are indicated by orange circles.

Aside from *K. pneumoniae* chromosomes, the *oqxAB-oqxR* genes are found on plasmids carried by *Enterobacteriaceae* hosts including *Escherichia coli*, *Shigella flexneri* and *Salmonella enterica* [6,9]. Given how readily chromosomal *oqxR* mutants can be selected by antibiotics in *K. pneumoniae*, it was of interest to investigate the conservation of *oqxAB-oqxR* in these plasmids, which may demonstrate high levels of diversity. There were a large number of non-*Klebsiella* genome sequences that contained the *oqxAB-oqxR* operon (*n*=321), so sequences were clustered based on similarity and sequences representative of the major clusters that differed from the *oqxAB-oqxR* structure previously reported for pOLA52 [7] were selected for inspection by amino acid sequence similarity graphs using Clinker (Fig. 3) . The *oqxR* regulator gene sequence was consistently found in plasmids carrying the pump, but the positive regulator gene *rarA* does not appear to have been mobilized onto plasmids. This may be due to RarA being involved in global regulation [10,11] and therefore carriage on plasmids may disrupt regular cellular function and reduce fitness.

Several *K. pneumoniae* strains were found to be carrying *oqxAB-oqxR* both on a plasmid and on the chromosome. In one instance, *K. pneumoniae* strain 555, the plasmid copy of *oqxAB* was frameshifted and may no longer be functional, but this may be a sequencing artefact (Fig. S1, available in the online version of this article). In other instances of co-carriage (strains KP32558 and Nord9 R85), the chromosomal and plasmid *oqxAB* sequences were highly similar (identity ≥99%) with no frameshift mutations, suggesting that both copies are functional. However, the *oqxR* gene on Nord9 pR85_1 is truncated, suggesting that the regulator is non-functional (Fig. S2). The *oqxR* gene in pR85_1 is adjacent to an IS*4* and a second copy of this insertion sequence is encoded upstream of the *oqxAB* genes. Therefore, the truncation of *oqxR* may have resulted from the actions of IS*4*, which may have mobilized the cluster, but only partially captured *oqxR*.

Comparisons at the amino acid level between chromosomally and plasmid encoded sequences of OqxR revealed that most (15/19) were identical to that of WT OqxR (Fig. S3). The chromosomally encoded OqxR in the Nord 9 strain (CP091582.1) has a single amino acid change, V130A, which may result in a similar resistance phenotype to the spontaneous mutants discovered in this study. As noted above, this strain also carries a plasmid with a truncated sequence of *oqxR* which, in combination with the point mutation in the chromosomal copy of *oqxR*, suggests that OqxAB expression may be high in this strain. The OqxR sequences for *K. pneumoniae* strain 555, *K. pneumoniae* KP32558 and pKP14052-MCR-1 (CP043932.1, CP076030.1 and MH715960.1) have an amino acid change at S15R which similarly may lead to *oqxAB* de-repression. Strain 555 carries a frameshifted plasmid copy of *oqxAB*.

Given that the majority of sequences for *oqxR* were identical to the WT sequence, it was of interest to determine if there were any differences in the promoter/operator regions between plasmid and chromosomal copies of the *oqxAB-oqxR* operon. We therefore examined the regions upstream of representative *oqxA* and *oqxR* loci to identify differences in their promoter/operator regions compared to the canonical chromosomal sequence. The sequences upstream of *oqxA* were identical between plasmid and chromosomal copies, suggesting that the binding sites for regulators and the transcription start site (TSS) are unaltered (Fig. S4).

In contrast, there were significant variations in the promoter regions of the *oqxR* loci. Each of the chromosomal sequences had the same promoter region, identical to that previously reported by Chan *et al*. [30] with a predicted TSS 62 bp upstream of the *oqxR* gene (Fig. 3a). The plasmid *oqxR* promoter/operator regions clustered into three groups based on sequence similarity, with two further sequences that did not fall into any of these groups. There was no correlation of groups with species origin, since plasmids from *E. coli*, *Salmonella enterica* and *K. pneumoniae* fell into each cluster, suggesting inter-species mobility. Many predicted *oqxR* promoter sequences on the plasmids matched those reported previously [30], but there were some distinct differences. Plasmids falling into the group with *E. coli* pOLA52 differed from the *Salmonella enterica* Typhimurium pHK06-53 grouping by a single nucleotide change C→T located 38 bases upstream of the *oqxR* start codon. This resulted in the promoter sequence predicted by BPROM to be upstream of the start codon, which would result in a full *oqxR* transcript being produced (Fig. 3a), unlike pHK06-53 [30]. Similarly, the BPROM predicted promoter sequence for *Salmonella enterica* Typhimurium pNDN5_LS002 was located well upstream of the *oqxR* start codon, which would also imply that the *oqxR* transcript produced would include the full-length *oqxR* gene. In most cases the predicted promoter sequences are yet to be verified experimentally, but these findings suggest that in at least some instances of plasmid carriage of the *oqxAB-oqxR* operon, the OqxR regulator could be fully functional.

Previous studies have suggested that *oqxAB-oqxR* can be mobilized by IS26-like transposases [9]. In the vast majority of plasmids *oqxAB-oqxR* are flanked by IS*26* in Tn*6010*. Tn*6010* was first identified in pOLA52 [8] and is found in a number of other sequences (Fig. 3b, grey stars). In one instance, Tn*6010* was present on the chromosome, in *K. pneumoniae* strain STEFF_2, which also had the native *oqx* operon intact (Fig. 3b, orange circles). *K. pneumoniae* STEFF_2 carries two plasmids, neither of which carries the *oqx* operon or IS*26*. Tn*6010* is not found in all plasmids carrying *oqxAB-oqxR*, suggesting these genes may have been acquired via an alternative element, or that multiple re-arrangement events have taken place modifying the initial Tn*6010* sequence. To highlight this diversity, a select number of representative sequences were included in Fig. 3b. In some plasmids ISs do flank *oqxAB-oqxR* but are not directly adjacent to the operon on one or both sides. Overall, the array of plasmids containing a copy of the operon is quite diverse, suggesting high levels of selection for *oqxAB-oqxR* carriage on plasmids.

## Discussion

While efflux pumps play an important role in intrinsic resistance and other cellular processes, it is typically their regulators that determine efflux pump expression levels, and thus determine their contribution to resistance to a particular antimicrobial [31]. Hypervirulent strains of *K. pneumoniae* NTUH-K2044, ATCC 43816 and SGH10 were included in our analysis as it was of interest to determine whether mutations relating to efflux upregulation would be selected in these strains, potentially increasing their multidrug resistance profile. The convergence of hypervirulent and multidrug-resistant *K. pneumoniae* strains, particularly in Asian regions, is of great concern [32,34]. Furthermore, mutations in *oqxR* have been highlighted as a potential contributor to the success of multidrug-resistant isolates of *K. pneumoniae* (e.g. Clonal Group 258) [12].

As demonstrated in this study, a wide range of mutations in *oqxR* can facilitate the switch from susceptible to resistant for a number of antibiotics. This is consistent with previous observations of mutations in *oqxR* leading to upregulation of *oqxAB* and subsequently increased resistance [4,11, 35]. Whether mutations in *oqxR* arise spontaneously under selective pressure or are present in the population at low frequencies and become enriched under selection is difficult to determine. There was no overlap between the mutations in *oqxR* found experimentally here and those previously reported, suggesting that effectively any mutation causing loss of function can be selected, rather than those in specific nucleotide locations. As such, *oqxR* mutation could be a simple genetic switch providing resistance in *K. pneumoniae*. It is important to report the breadth of mutations in regulators that can lead to increased levels of resistance, as this is fundamental to increasing the reliability of predicting resistance profiles from whole genome sequencing [35].

Whereas intrinsic *oqxAB* expression can provide low to intermediate levels of resistance, overexpression of *oqxAB* is required for strains to be considered non-susceptible, especially to ciprofloxacin [30,36]. Increased efflux pump expression can be associated with a fitness cost, although modifications in metabolic pathways can compensate for the negative effects of efflux pump overexpression [37]. We did not observe any significant fitness cost for *oqxR* mutations as measured by growth curves compared to the WT, but this was in rich lab media and may not reflect the environmental conditions of an active infection. A clinical isolate of *K. pneumoniae* with a mutation in *oqxR* resulting in upregulation of OqxAB was more virulent in a *Caenorhabditis elegans* model compared to the same strain without a mutation in *oqxR*, suggesting that fitness of the OqxAB overproducing strain was not significantly impacted [4]. Given the widespread dispersion of *oqxAB-oqxR* onto various plasmids, this would suggest that there may be selective pressure to maintain the efflux pump, and therefore the benefit must outweigh any potential cost.

It is possible that not all of the resistant mutants that were observed in the replica plating experiments harboured mutations in *oqxR*. For instance, mutations in the quinolone resistance-determining regions of *gyrA*, *gyrB*, *parC* and *parE* are commonly associated with resistance to ciprofloxacin [38]. However, these mutations would not confer resistance to either tetracycline or chloramphenicol and the majority of colonies when replica plated were able to survive on all three antibiotics, suggesting a non-specific mechanism of resistance. Furthermore, mutations in topoisomerase enzyme genes must occur within a relatively restricted region and at specific sites to maintain function of the enzyme but prevent quinolone binding. In contrast, a mutation at almost any site in *oqxR* that introduces a frameshift or changes a functionally important amino acid residue in the encoded protein will allow for increased *oqxAB* expression and therefore a change in resistance.

While the *oqxAB-oqxR* operon was frequently identified on plasmids and found to be highly conserved (nucleotide sequence identity >99%), significant diversity was identified in the promoter region upstream of *oqxR*. Chan *et al*. [30] identified that in *Salmonella enterica* subsp. Typhimurium strain PY1, expression of the *oqxR-oqxAB* operon cloned from the plasmid pHK06-53 led to increased resistance to ciprofloxacin. In contrast, expression of *oqxR-oqxAB* cloned from the chromosome of *K. pneumoniae* strain MGH78578 did not result in increased ciprofloxacin resistance. They identified that the promoter region of *oqxR* was truncated in the pHK06-53 plasmid-borne copy of the *oqxR-oqxAB* operon and showed that this resulted in transcription of *oqxR* from an alternative start site that reduced its level of expression and shortened the OqxR protein sequence. This reduced its binding capacity as a negative regulator, which in turn reduced repression of the *oqxAB* operon [30]. The authors hypothesized that this truncation was the result of the promoter being partially captured in a transposon, Tn*6010* [30]. The protein sequence of the truncated OqxR is yet to be experimentally verified, but if the predicted TSS is correct, this would lead to the omission of at least 12 aa from the N-terminus of the protein. It is interesting to note that the alternative promoter and TSS proposed by Chan *et al*. [30] is present in both chromosomal and plasmid-based *oqxR* sequences (Fig. 3a), so could also direct transcription in the native chromosomal context. Presumably, it would not be beneficial to produce full-length and truncated versions of OqxR from the chromosomally encoded operon, so there may be additional unknown mechanisms of regulation to prevent this.

In the current work, we found evidence to suggest that at least some plasmid sequences of *oqxR*, including pNDM5_LS002 found in *Salmonella enterica* subsp. Typhimurium, could produce a full-length transcript and subsequently fully functional OqxR. Furthermore, some sequences that contained Tn*6010*, such as *E. coli* pOLA52, *E. coli* pWP2-W18-CRE-03_1 and *K. pneumoniae* STEFF_2, had predicted promoters that would also produce a full-length *oqxR* transcript due to a single point mutation in the region between IS*26* and the *oqxR* start codon. This raises questions around the regulation and expression level of the OqxAB pump in these circumstances and what the contribution to resistance would be.

There were a number of *K. pneumoniae* strains that carried both a chromosomal and plasmid encoded copy of the *oqxAB-oqxR* operon. One study that investigated the presence of *oqxAB* in ESBL (extended-spectrum beta-lactamase) *K. pneumoniae* strains found that co-carriage of *oqxAB* on both the chromosome and plasmids occurred in 13% of isolates tested (*n*=114) [36]. The reason for this is unclear, but it has been shown that expression of *oqxAB* from plasmids can increase by >80-fold [9], so this may be an alternative way of gaining resistance from OqxAB expression without selecting for mutations in the chromosomally located *oqxR* gene. It is also possible that regulators from the chromosomally encoded operon could interact with the plasmid copy of *oqxAB*, or vice versa. Additionally, co-carriage could provide a source for recombination to interchange chromosomal and plasmid copies of the operon. This could enable the fine-tuning of OqxAB expression in response to environmental challenges.

The inherent danger of efflux pumps to infection control is that a single mutation can lead to broad resistance to a range of antimicrobials. The *oqxR* regulator clearly plays an important role in determining the resistance profile associated with *oqxAB* pump expression. In essence, the OqxAB pump has a fairly similar range of substrate recognition to that of AcrAB, an RND pump broadly present in *Enterobacteriaceae*, the primary difference being that *acrAB* is carried natively in the chromosome of most lineages, whereas *oqxAB* is generally restricted to *K. pneumoniae* and *Enterobacter* sp. [9]. To date, there is no documented carriage of *acrAB* on plasmids, but there is clear evidence of mobilization of *oqxAB* broadly on plasmids. It is interesting that a pump that presumably only has utility when combined with a TolC outer membrane component would be mobilized and transferred between strains on a plasmid, and could potentially explain the limited host range of the *oqxAB* operon as it may only have utility in strains that possess TolC. It would be interesting to know if OqxAB competes with AcrAB for TolC attachment. Indeed, membrane facilitator proteins from various multi-component efflux transporters have been shown to have differing affinities for TolC, probably contributing to the recruitment and stability of pump-TolC complexes [39]. Both OqxAB and AcrAB have been shown to contribute to tigecycline, nitrofurantoin and ciprofloxacin resistance in *K. pneumoniae* [40,42]. The interplay between these efflux pumps warrants further investigation, and the spread of *oqxAB* should be monitored due to its potential contribution to multidrug resistance.

## supplementary material

10.1099/mic.0.001499 Supplementary Material 1.

## Abbreviations

AMR: antimicrobial resistance
CFU: colony forming unit
LLB: Lennox-LB
MH: Mueller-Hinton
MIC: minimal inhibitory concentration
PBS: phosphate buffered saline
RND: resistance nodulation division
TSS: transcription start site
WHO: World Health Organisation
WT: wild type
