# Supplementary material for: Mutations in the efflux regulator gene oqxR provide a simple genetic switch for antimicrobial resistance in Klebsiella pneumoniae
Source: Microbiology (Reading). 2024 Sep 4;170(9):001499. doi: 10.1099/mic.0.001499 (PMC11373524; doi:10.1099/mic.0.001499)
Supplement: Supplementary Material 1. [file mic-170-01499-s001.pdf]

OqxR-oqxAB Supplementary info

Table S1. Strains used in the study

Species	Strain	Strain designation	Source
Klebsiella pneumoniae	KP6870155	KH241	(Semenec et al., 2023)
	SGH10	KH373	(Lam et al., 2018)
	ATCC43816	KH372	(Short et al., 2020)
	NTUH-K2044	KH374	
	ATCC43816 C.1	KH406	This study – *oqxR* mutants
	ATCC43816 C.2	KH407	
	SGH10 C.1	KH408	
	SGH10 C.2	KH409	
	NTUH-K2044 C.1	KH410	
	NTUH-K2044 C.2	KH411	
	KP6870155 C1.1	KH412	
	KP6870155 C1.2	KH413	
	KP6870155 C2.3	KH414	
	KP6870155 C2.4	KH415	
	KP6870155 C3.1	KH416	
	KP6870155 C3.2	KH417	

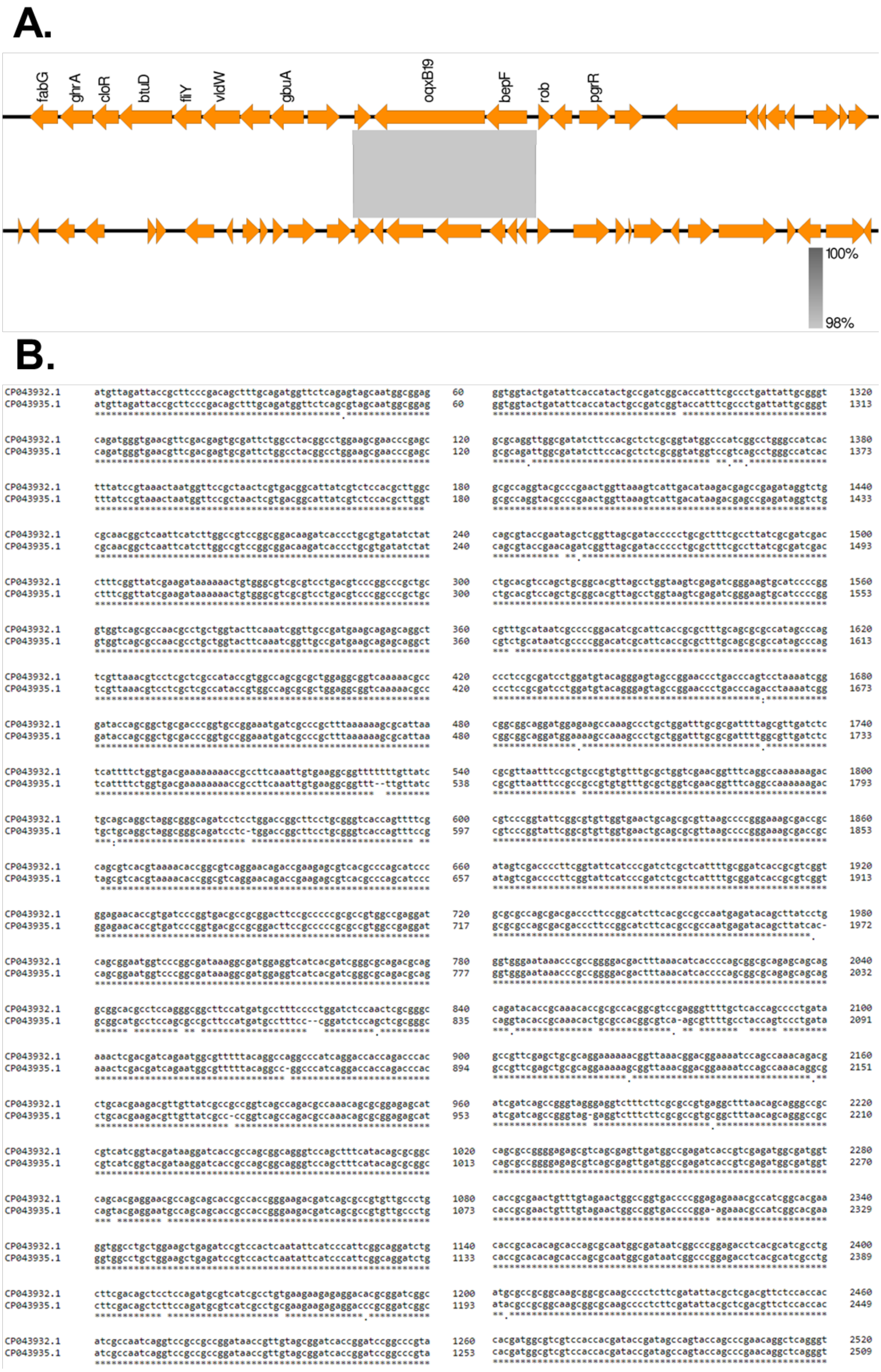
A.
fabG
ghrA
cloR
btuD
fliY
vldW
gbuA
oqxB19
bepF
rob
pgrR
100%
98%
B.
CP043932.1
CP043935.1

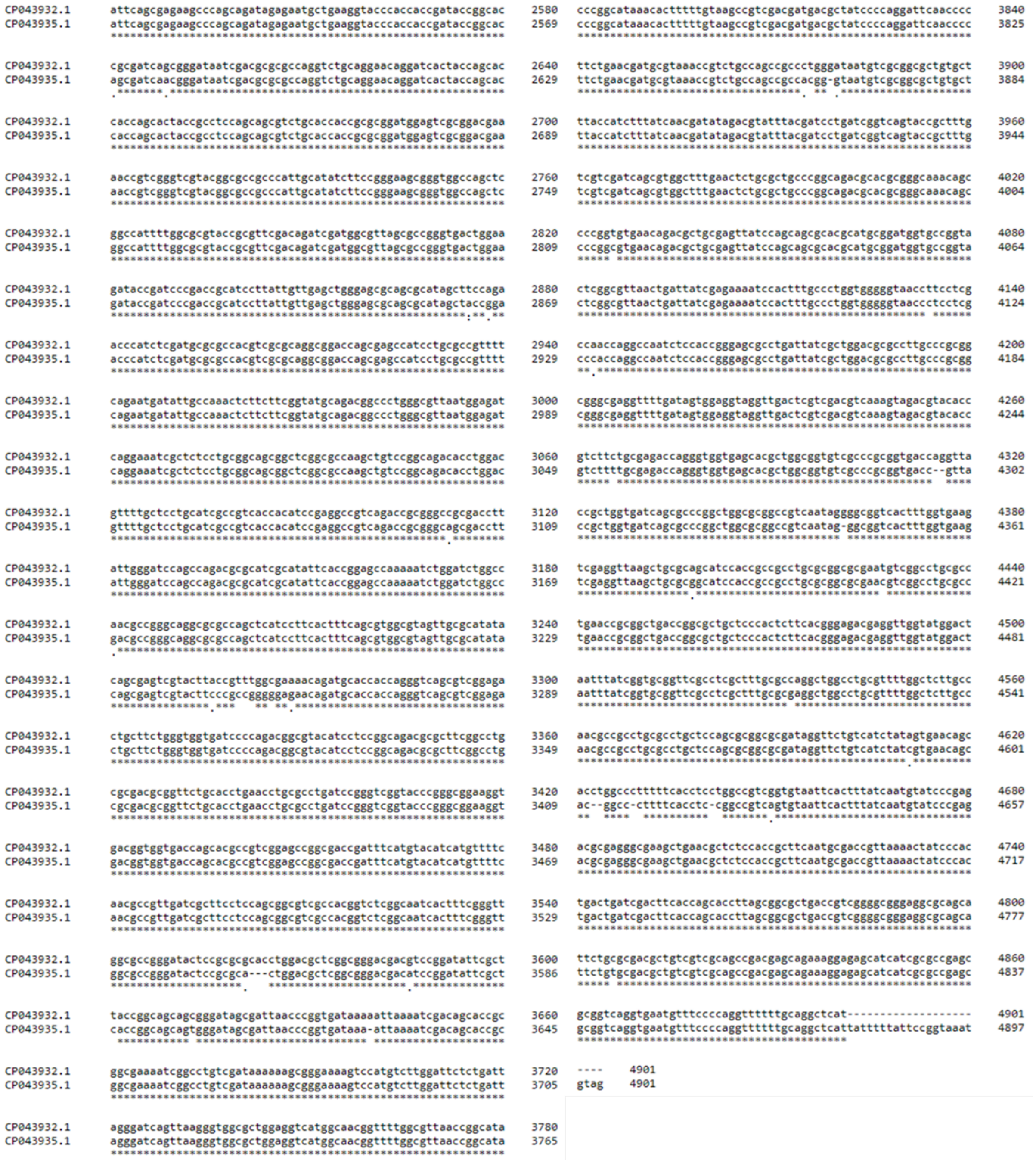

Figure S1. Comparison of sequences *K. pneumoniae* 555 (CP043932.1, top) and K. pneumoniae 555 pSCKLB555-3 (CP043935.1, bottom) **A.)** Graphical comparison at the nucleotide level, created using Easyfig v 2.2.2 and blast v 2.7.1 (Sullivan et al., 2011). *K. pneumoniae* 555 is the top strand and K. pneumoniae 555 pSCKLB555-3 the bottom strand **B.)** Sequence alignment using Clustal Omega v 1.2.4 (Sievers and Higgins, 2018). Sequence starts at *oqxR* and is aligned to *oqxA* (annotated as *bepF* in panel A).

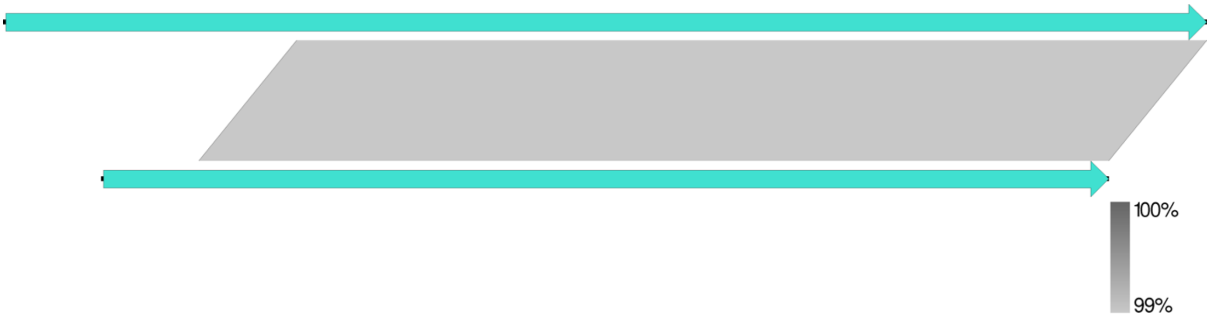

Figure S2. Comparison of *oqxR* sequences from *K. pneumoniae* Nord9 R85 (CP091582.1, top) and Nord 9 R85 plasmid pR85_1 (CP091583.1, bottom) at the nucleotide level using Easyfig. The truncation may be due to partial capture of the sequence by an IS4-like transposase that is encoded upstream of *oqxR.*

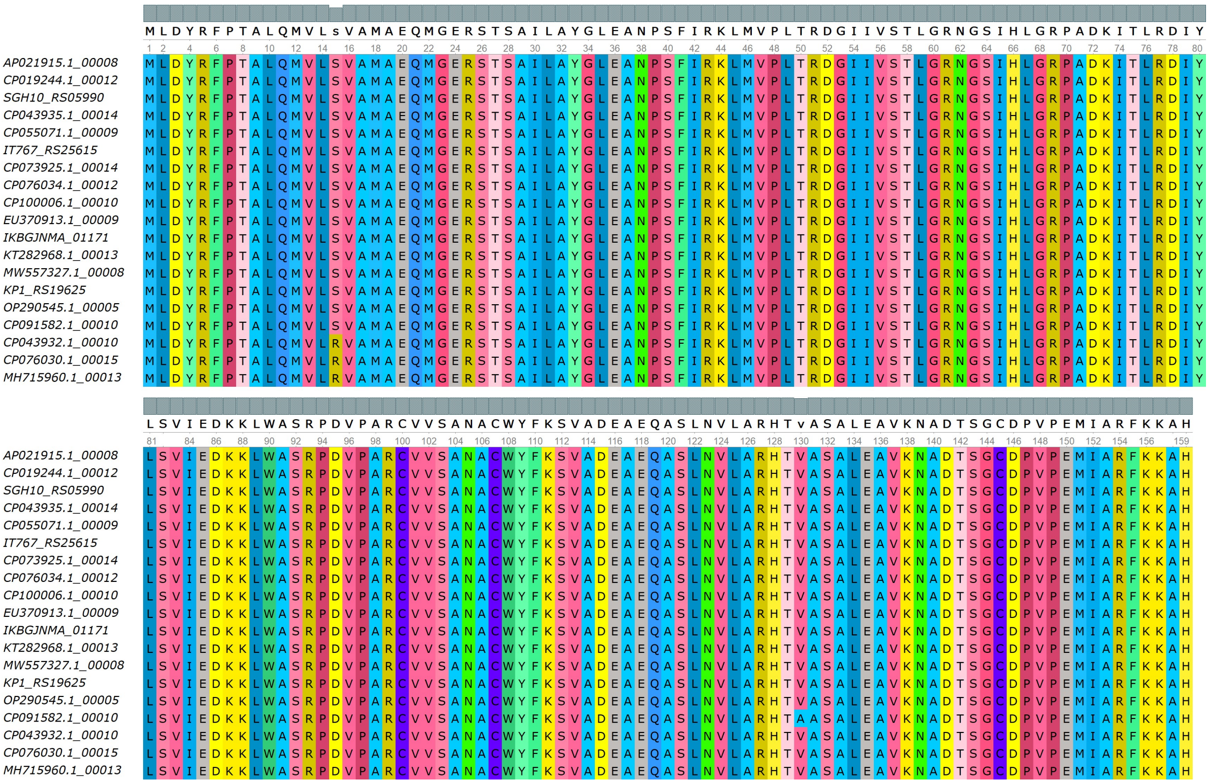

Figure S3. Comparison of OqxR at the amino acid level. Alignments performed using MAFFT and visualised in UGENE.

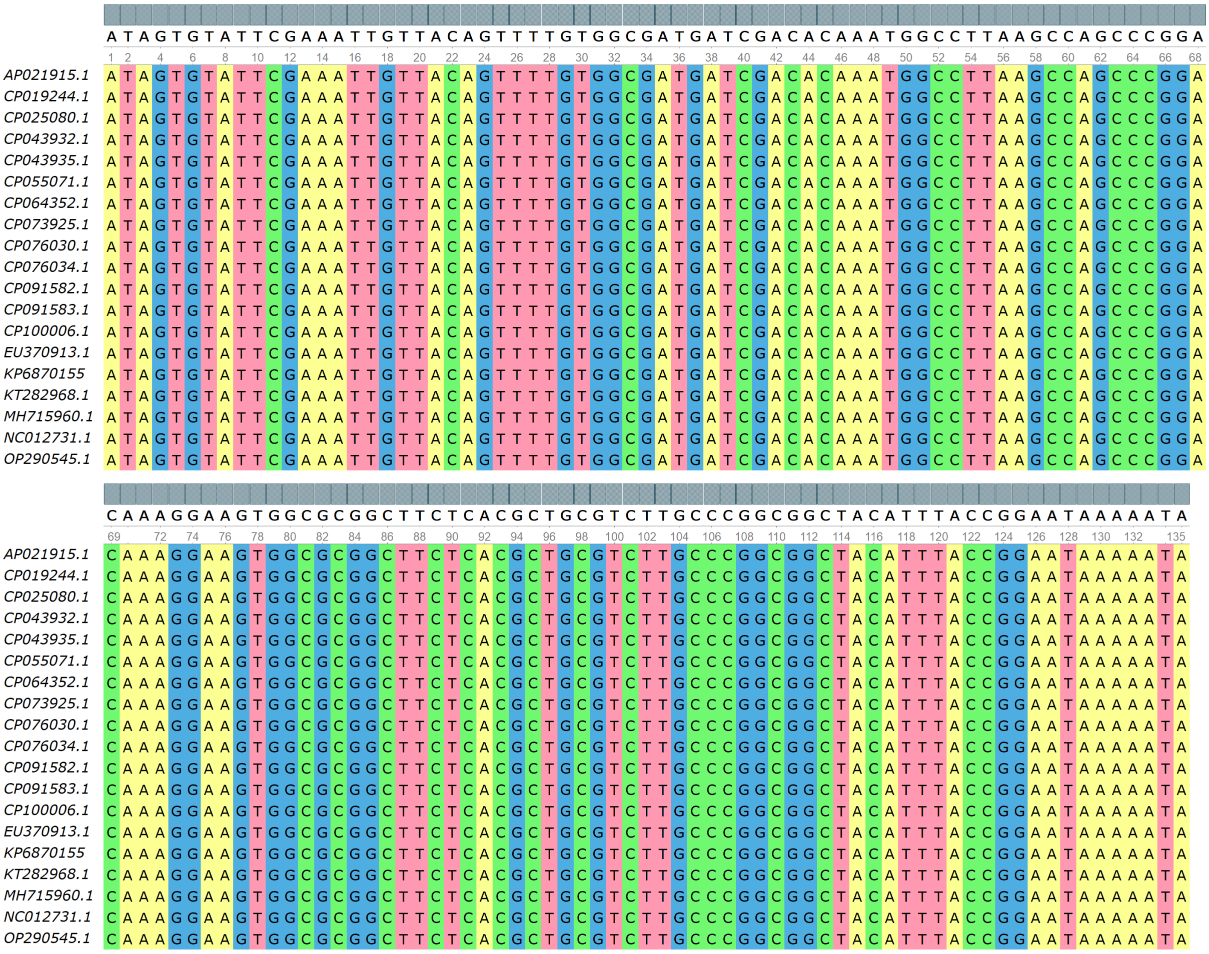

Figure S4. Comparison of *oqxA* promoter region. Sequences were aligned using MAFFT and visualised using UGENE.

References

Lam, M. M. C., Wyres, K. L., Duchêne, S., Wick, R. R., Judd, L. M., Gan, Y.-H., Hoh, C.-H., Archuleta, S., Molton, J. S., Kalimuddin, S., et al. (2018). Population genomics of hypervirulent Klebsiella pneumoniae clonal-group 23 reveals early emergence and rapid global dissemination. *Nat Commun* 9, 2703.

Semenec, L., Cain, A. K., Dawson, C. J., Liu, Q., Dinh, H., Lott, H., Penesyan, A., Maharjan, R., Short, F. L., Hassan, K. A., et al. (2023). Cross-protection and cross-feeding between Klebsiella pneumoniae and Acinetobacter baumannii promotes their co-existence. *Nat Commun* 14, 702.

Short, F. L., Di Sario, G., Reichmann, N. T., Kleanthous, C., Parkhill, J. and Taylor, P. W. (2020). Genomic Profiling Reveals Distinct Routes To Complement Resistance in Klebsiella pneumoniae. *Infect Immun* 88, e00043-20.

Sievers, F. and Higgins, D. G. (2018). Clustal Omega for making accurate alignments of many protein sequences. *Protein Science* 27, 135–145.

Sullivan, M. J., Petty, N. K. and Beatson, S. A. (2011). Easyfig: a genome comparison visualizer. *Bioinformatics* 27, 1009–1010.
